# Clinical efficacy and mechanisms of transcutaneous auricular vagus nerve stimulation targeting the gut-brain axis for postoperative complications of aortic dissection: study protocol for a randomized controlled trial

**DOI:** 10.3389/fmed.2025.1692356

**Published:** 2025-12-18

**Authors:** Bo Ning, Liangbin Yang, Yi Wei, Cheng Luo, Feisheng Zheng, Teng Ge, Haining Ou, Chaojie Wang, Jinlin Hu, Qingzuo Zhao, Jingyu Bo, Kai Wang, Zhan Zhang, Hongyu Chen, Rongjun Zou, Xiaoping Fan, Jihai Peng

**Affiliations:** 1 The Second Clinical Medical College, Guangzhou University of Chinese Medicine, Guangzhou, China; 2 The Fourth Clinical Medical College, Guangzhou University of Chinese Medicine, Shenzhen, China; 3 Clinical Medical College, Chengdu University of Traditional Chinese Medicine, Chengdu, China; 4 Guangdong Provincial Hospital of Chinese Medicine, The Second Affiliated Hospital of Guangzhou University of Chinese Medicine, Guangzhou, China; 5 The First Clinical Medical College, Shaanxi University of Chinese Medicine, Xianyang, China; 6 State Key Laboratory of Traditional Chinese Medicine Syndrome, State Key Laboratory of Dampness Syndrome of Chinese Medicine, Guangdong Provincial Key Laboratory of TCM Emergency Research, Guangzhou, China

**Keywords:** transcutaneous auricular vagus nerve stimulation, gut-brain axis, aortic dissection, postoperative complications, randomized controlled trial

## Abstract

**Background:**

Aortic dissection (AD) is a life-threatening cardiovascular emergency characterized by rapid onset and high mortality. While surgery intervention, the primary treatment, improves short-term survival, it frequently leads to postoperative complications including systemic inflammatory response syndrome, gastrointestinal dysfunction, and anxiety/depression. These complications may be exacerbated by dysregulation of the gut-brain axis (GBA). Transcutaneous auricular vagus nerve stimulation (taVNS) is a non-invasive neuromodulation technique known to exert anti-inflammatory, prokinetic, and neuroregulatory effects in various conditions; however, its application for managing postoperative complications in AD remains unexplored. This study aims to evaluate the efficacy and safety of taVNS in regulating the GBA among postoperative AD patients through a randomized controlled trial.

**Methods:**

This is a single-center, randomized, investigator-blinded, sham-controlled randomized controlled trial. A total of 50 patients aged 18–75 years with postoperative Stanford Type A or B AD will be enrolled and randomly assigned in a 1:1 ratio to either the active taVNS group or the sham control group. Both groups will receive standard postoperative care. The experimental group will additionally receive active taVNS targeting the vagus nerve-innervated auricular area (15 Hz, 200 μs pulse width), while the control group will receive sham stimulation at a non-vagus innervated site without electrical current. The intervention will be administered for 30 min, twice daily, over 7 consecutive days, with follow-up assessments continuing until 24 weeks post-surgery. Primary outcomes include changes in gut microbiota diversity/abundance and brain function (assessed via functional near-infrared spectroscopy). Secondary outcomes encompass inflammatory markers, plasma neurotransmitter levels, intestinal function recovery, and relevant psychometric scale scores. Safety will be monitored through vital signs, laboratory tests, and recording of any adverse events.

**Discussion:**

This study is the first to innovatively integrate taVNS with the GBA theory, investigating its multi-target mechanisms through a comprehensive set of biomarkers and clinical endpoints. If proven effective, taVNS could offer a safe and cost-effective non-pharmacological adjunctive therapy for managing postoperative complications in AD. Furthermore, the findings have the potential to elucidate the role of the GBA in the recovery trajectory of patients following major cardiovascular surgery.

**Trial Registration:**

Chinese Clinical Trial Registry (ChiCTR, http://www.chictr.org.cn), No. ChiCTR2500102345, Date: May 13, 2025.

## 1 Introduction

Aortic dissection (AD) is a life-threatening cardiovascular emergency characterized by a tear in the aortic intima, leading to blood flow within the aortic wall and formation of a false lumen (1). AD is classified as Stanford Type A (involving the ascending aorta) or Type B (confined to the descending aorta). Untreated Type A AD carries a mortality rate exceeding 50% within 48 h (2, 3). The incidence of acute AD is approximately 1.9-fold higher in men than in women, with a male-to-female ratio of 2:1–3:1 for Stanford Type A AD (3). The pathophysiology of AD primarily involves structural abnormalities of the aortic wall, hemodynamic stress, and inflammatory activation (4–6). The gut-brain axis (GBA) offers a novel perspective for intervening in multi-system diseases. Gut microbiota dysbiosis is implicated in AD pathogenesis, exacerbating vascular inflammation and remodeling via the GBA (7). Furthermore, dysbiosis and impaired intestinal barrier function can amplify systemic inflammatory response syndrome (SIRS) through neural, immune, and endocrine pathways, adversely affecting cardiovascular and central nervous system function (8).

While surgery significantly improves short-term survival in AD, postoperative complications—including SIRS, gastrointestinal dysfunction, anxiety, and depression—remain major challenges that adversely impact long-term prognosis (9). These complications are closely linked to GBA dysregulation. Intestinal dysbiosis promotes vascular inflammation via neural, immune, and endocrine pathways, while gut barrier dysfunction fuels SIRS, creating a vicious cycle with neuropsychological impairment (10). Despite advances in acute AD management, effective long-term strategies are lacking. The critical postoperative status of AD patients in the ICU—with unstable vital signs, impaired consciousness, and polypharmacy—poses a major challenge for clinical research. Existing therapies often fail to concurrently address cardiovascular instability, inflammation, and quality of life, particularly in patients with comorbid psychological stress and intestinal dysfunction. Moreover, the impact of conventional treatments on the GBA is unknown, underscoring the need for novel interventions.

Transcutaneous auricular vagus nerve stimulation (taVNS) is a non-invasive technique that modulates autonomic nervous system activity, suppresses pro-inflammatory cytokine release, and improves intestinal and central neurotransmitter function by stimulating the auricular branch of the vagus nerve (11). However, its application in post-surgical AD patients remains unexplored. taVNS enhances vagal tone, mitigates SIRS, and provides cardioprotection (12). It also restores intestinal barrier integrity by modulating gut microbiota composition—increasing beneficial genera like *Bifidobacterium* and *Lactobacillus*—and reducing pathogenic metabolites (13). Additionally, taVNS alleviates bloating and constipation by enhancing vagal efferent signaling, which promotes gastrointestinal motility and digestive secretion (14). In AD patients, these effects could reduce gut-derived endotoxin absorption and lower systemic inflammation. Although gut microbiota dysregulation and GBA disorders are evident in AD, this field is still emerging. More foundational research is required to definitively link taVNS with GBA modulation post-AD and establish it as a therapeutic target.

Based on previous studies, taVNS offers the following advantages for intervening in postoperative complications of AD: ① Its non-invasive nature avoids the risks of invasive procedures, making it suitable for the long-term postoperative management of AD patients; ② It enables multi-target regulation of the autonomic nervous system, inflammatory response, and intestinal function; ④ Existing clinical trials show that taVNS has mild side effects, no serious complications, and high safety.

Therefore, we hypothesize that taVNS can ameliorate systemic inflammation, enhance intestinal and neurological recovery, stabilize hemodynamics, and reduce complications in postoperative AD patients by modulating the GBA. We have designed a randomized controlled trial to evaluate the clinical efficacy and mechanisms of taVNS, focusing on GBA regulation. This study is the first to investigate taVNS in AD patients, addressing the evidence gap for targeted GBA interventions after cardiovascular surgery.

## 2 Objectives

This study aims to evaluate the efficacy and safety of taVNS on GBA-related outcomes in postoperative AD patients. Key endpoints include cardiovascular function, hemodynamics, inflammatory status, gut microbiota, neurotransmitter levels, psychological scores, and quality of life. Furthermore, we seek to provide evidence for the role of taVNS in regulating the GBA to mitigate postoperative complications in AD.

## 3 Methods

### 3.1 Design

**Figure 1:**
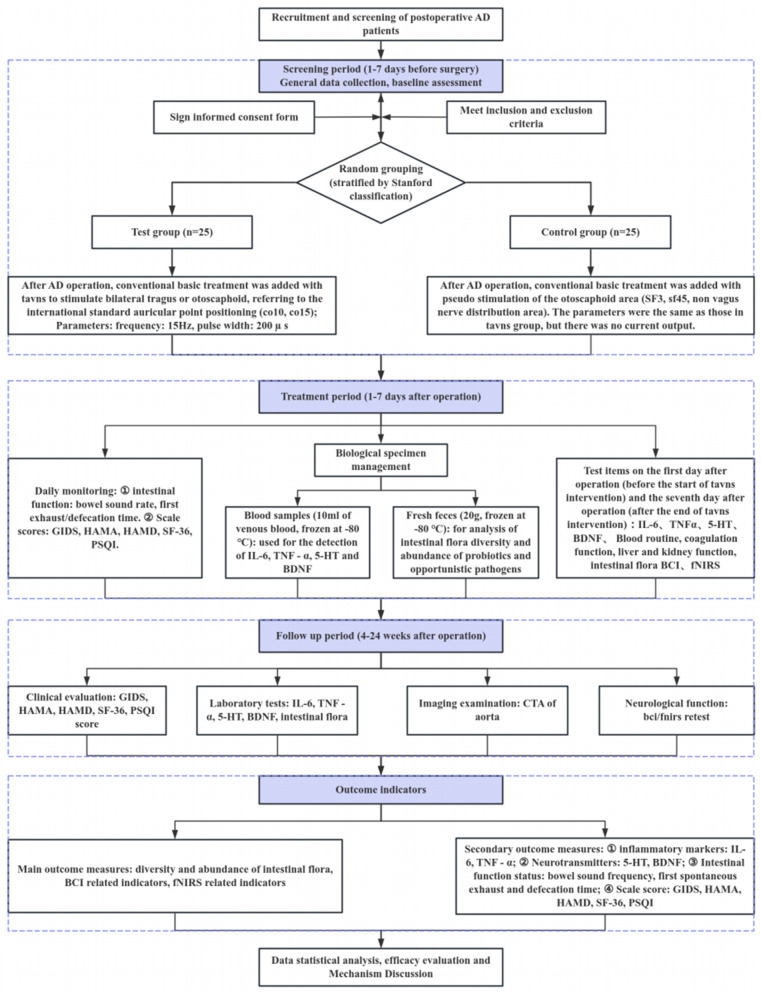
Clinical trial flow chart.

This is a single-center, randomized, investigator-blinded, sham-controlled trial. The protocol was registered with the Chinese Clinical Trial Registry (ChiCTR2500102345) on 13 May 2025 and approved by the Ethics Committee of Guangdong Provincial Hospital of Traditional Chinese Medicine (Approval No. ZF2025-162-01) on 18 April 2025. The study will be conducted in accordance with the Declaration of Helsinki. A total of 50 eligible postoperative AD patients will be recruited from the hospital and randomized to receive either active taVNS or sham stimulation. The study flow is outlined in Figure 1, and the schedule of events is presented in Table 1.

**Table 1:** Schedule of clinical trial index collection.

**Indicators**	**Screening period**	**Treatment period**			**Follow-up period**	
	**Visit 1 (days 1-7 before surgery)**	**Visit 2 (postoperative day 1, before intervention)**	**Visit 3 (daily monitoring for 1-7 days post-intervention)**	**Visit 4 (7 days post-intervention)**	**Visit 5 (4 weeks after surgery)**	**Visit 6 (24 weeks after surgery)**
General Information	✓					
Physical Examination	✓	✓	✓	✓	✓	✓
Written informed consent	✓					
Blood routine testduplicate	✓	✓		✓		
Liver function	✓	✓		✓		
Renal function	✓	✓		✓		
Coagulation function	✓	✓		✓		
Urine routine test	✓					
Urine pregnancy test in women of childbearing age	✓					
CTA of the Aorta	✓				✓	✓
Frequency of bowel sounds		✓	✓	✓		
The time of first flatus/defecation		✓	✓	✓		
GIDS		✓	✓	✓	✓	✓
HAMA		✓	✓	✓	✓	✓
HAMD		✓	✓	✓	✓	✓
SF-36		✓	✓	✓	✓	✓
PSQI		✓	✓	✓	✓	✓
IL-6		✓		✓	✓	✓
TNF-α		✓		✓	✓	✓
5-HT		✓		✓	✓	✓
BDNF		✓		✓	✓	✓
Gut Microbiota		✓		✓	✓	✓
BCI		✓		✓	✓	✓
NIRS		✓		✓	✓	✓
Adverse events	Record at any time					
Review of CRF	Review at any time					

GIDS, gastrointestinal dysfunction score in critically ill patients; HAMA, Hamilton anxiety scale; HAMD, Hamilton depression scale; SF-36, the short form 36 health survey; PSQI, Pittsburgh sleep quality index; IL-6, interleukin-6; TNF-α, tumor necrosis factor-α; 5-HT, 5-hydroxytryptamine; BDNF, brain-derived neurotrophic factor; BCI, brain-computer interface; NIRS, near infrared spectroscopy.

### 3.2 Participants

#### 3.2.1 Diagnostic criteria for AD

The diagnostic criteria for AD were established with reference to the *Thoracic Surgery Clinical Practice Guidelines on the Management of Type B Aortic Dissection* (STS/AATS, 2022) (15) and the *Guideline for the Treatment of Thoracic Aortic Dissection Type A* (jointly released by multiple German societies, 2022) (16). They are specified as follows: ① Typical symptoms: Sudden, severe tearing or ripping pain in the chest or back, which may radiate to the abdomen, lower extremities, or back; some patients may present with no obvious pain (e.g., those with Marfan syndrome). ② Typical signs: A systolic blood pressure difference of >20 mmHg between the two upper limbs; a diastolic murmur audible over the aortic valve area; disturbances of consciousness; or signs of organ malperfusion (e.g., oliguria, lower limb pallor). ③ Imaging examination: Computed tomography angiography confirms the diagnosis by revealing an aortic intimal tear and the formation of true and false lumens. ④ Stanford classification criteria: Type A: Dissection involves the ascending aorta; Type B: Dissection is confined to the descending aorta.

#### 3.2.2 Inclusion criteria

① Patients with postoperative Stanford Type A or Type B AD who are hemodynamically stable;

② Aged 18–75 years (inclusive), regardless of gender;

③ Have voluntarily provided a signed written informed consent form.

Only subjects who meet all three criteria above are eligible for enrollment in this study.

#### 3.2.3 Exclusion criteria

① Severe hepatic or renal dysfunction: estimated glomerular filtration rate < 30 mL/min/1.73 m^2^ or Child-Pugh Class C;

② History of major psychiatric or cognitive disorders;

③ Pregnancy, lactation, or a positive urine pregnancy test at screening;

④ Resting heart rate < 50 bpm or resting systolic blood pressure < 90 mmHg;

⑤ Severe active infections, autoimmune diseases, chronic gastrointestinal diseases, or use of antibiotics/probiotics within 4 weeks prior to enrollment;

⑥ Concurrent use of medications that significantly affect autonomic nervous function (e.g., beta-blockers, anticholinergic drugs);

⑦ Contraindications to taVNS, such as known skin allergy to electrode materials or active ear infection;

⑧ Considered by the investigator to be unsuitable for trial participation.

Subjects meeting any of the above criteria will be excluded.

#### 3.2.4 Removal criteria

Enrolled subjects will be removed from the study if any of the following conditions are met: ① Poor compliance, defined as a taVNS intervention completion rate of < 80%, or explicit refusal to cooperate with study procedures; ② Missing key outcome data (e.g., inflammatory markers, gut microbiota data) that preclude efficacy assessment. Reasons for removal will be documented in detail, and the case report form (CRF) will be retained. Data from removed subjects will be excluded from the per-protocol efficacy analysis but included in the safety analysis if they received at least one taVNS intervention.

#### 3.2.5 Drop-out criteria

Investigator-determined withdrawal: ① Occurrence of a serious adverse event (SAE) leading to trial discontinuation based on investigator judgment; ② Development of a new severe intercurrent illness that may confound efficacy or safety assessments.

Subject-initiated withdrawal: ① Voluntary withdrawal from the trial for any reason; ② Loss to follow-up, defined as failing to receive the intervention or complete assessments for two consecutive scheduled visits despite attempts to make contact.

Handling of Drop-out Cases: ① The investigator will attempt to contact the subject to document the reason for dropout and the last intervention date, and to complete any feasible assessments; ② Subjects who dropout due to adverse events will receive appropriate clinical management; ③ All data from dropout cases will be preserved and included in the intent-to-treat (ITT) analysis.

### 3.3 Sample size estimation

The sample size for this study was calculated using the standard formula for comparing means between two independent groups in a randomized controlled trial. The formula and parameters are defined as follows: Sample Size Calculation Formula:


$$\begin{array}{c} n=2\times {\left[\frac{\left(Z_{\alpha /2}+Z_{\beta /2}\times \sigma \right)}{\Delta }\right]}^{2} \end{array}$$ (1)

Where: *n* = sample size per group; *Z*_α_/2 = critical value for a two-tailed test at significance level α; *Z*_β_ = critical value for Type II error rate β; σ= population standard deviation of the primary outcome; Δ = minimum clinically important difference.

Basis for Parameter Setting: *Z*_α_/2: A two-tailed significance level (α) of 0.05 was used, corresponding to *Z*_α_/2 = 1.96; *Z*_β_: A statistical power (1 – β) of 80% was targeted (β = 0.2), corresponding to *Z*_β_ = 0.84; Δ and σ: Based on previous taVNS randomized controlled trials (17–19) and our pilot data, we assumed a continuous primary outcome (e.g., postoperative quality of life score or change in serum IL-6). The expected between-group difference (Δ) was three points, with a common standard deviation (σ) of five points.

Sample Size Calculation Result: Using the above parameters, 22 participants were required per group, yielding an initial total sample size of 44. Accounting for an anticipated 10% dropout rate, the final sample size was increased to 50 participants. These will be randomly assigned at a 1:1 ratio to the intervention (taVNS, *n* = 25) and control (sham stimulation, *n* = 25) groups.

Power analysis was performed for the primary outcomes (gut microbiota and brain function). The final sample size ensures ≥80% power for both. This sample size also covers the secondary endpoints and no separate calculation was needed.

### 3.4 Criteria for randomization and blinding

Stratified block randomization will be used, stratified by Stanford type (A or B). Random sequences were generated using SAS 9.4 software and concealed in sequentially numbered, opaque, sealed envelopes, which will be managed by an independent statistician.

A single-blind design (participant-blinded) is employed to minimize participant bias in self-reported outcomes (e.g., pain or bloating scores). The stimulation devices for both groups are identical in appearance. Each device has pre-programmed “active” and “sham” stimulation modes, which can only be toggled by a device engineer using a hidden password. In sham mode, the device displays identical operation indicators and parameter values as in active mode, but without any current output, thus preventing physiological effects and maintaining blinding. Outcome assessors for psychological scales (e.g., HAMA, HAMD, and GIDS) will be blinded to group assignment and not involved in intervention delivery. They will only receive de-identified subject IDs and corresponding raw scale data. Similarly, all samples for gut microbiota, serum biomarkers, and brain function analyses will be de-identified. Laboratory personnel will submit results linked only to sample codes, and the final linkage of codes to group assignment will be performed by the independent statistician.

### 3.5 Intervention

#### 3.5.1 Intervention group

Participants will receive conventional postoperative basic care for AD (e.g., blood pressure control, anti-infection therapy, and nutritional support) combined with taVNS: ① Stimulation sites: Bilateral tragus or cymba conchae, corresponding to the international standardized auricular points CO10 (“Shenmen”) and CO15 (“Cymba Conchae”), which are densely innervated by the auricular branch of the vagus nerve. ② Frequency = 15 Hz; pulse width = 200 μs; current intensity will be individually titrated from 1 mA to a perceptible yet non-painful level (maximum ≤ 5 mA). ③ taVNS stimulators certified by the National Medical Products Administration or the U.S. Food and Drug Administration.

#### 3.5.2 Control group

Participants will receive conventional postoperative care for AD (identical to the intervention group) plus sham stimulation: ① Stimulation target: The scaphoid fossa (auricular points SF3, SF5), a non-vagus innervated region. ② Electrode placement: Identical in appearance to the active group (bilateral tragus or cymba conchae) to maintain blinding. ③ Parameters: Frequency, pulse width, and duration match the active taVNS group, but with zero current output.

#### 3.5.3 Intervention and follow-up period

All patients will receive standardized postoperative management, including nutritional support (standard liquid/semi-liquid diet) supervised by the hospital nutrition department. Concomitant medications affecting gut microbiota or motility will be documented. ① Intervention frequency: Twice daily, 30 min per session. ② Start time: Postoperative day 1. ③ Intervention course: 7 consecutive days. ④ Follow-up: Participants will be followed for 24 weeks, with periodic assessments of efficacy (e.g., inflammatory markers, gut microbiota, quality of life) and safety (e.g., adverse events, vital signs).

### 3.6 Outcome measures

#### 3.6.1 Main outcome measures

① Gut Microbiota Diversity and Abundance: Fecal samples will be analyzed using metagenomic sequencing. Key metrics will include the abundance of probiotics (e.g., Bifidobacterium, Lactobacillus)—where reduced abundance reflects gut microbiota imbalance and indicates dysbiosis—the abundance of opportunistic pathogens (e.g., Escherichia coli, Enterococcus)—whose excessive proliferation may disrupt the intestinal barrier and induce systemic inflammation—and diversity indices (the Shannon index, which integrates species richness and evenness, with higher values indicating greater diversity; and the Simpson index, which emphasizes the dominance of key species, with lower values indicating greater diversity). These metrics will be used to evaluate the severity of postoperative gut microbiota dysbiosis in AD patients and the regulatory effects of taVNS on the microbiota.

② Brain Function Changes:

a. BCI-Related Metrics: Electroencephalography (EEG) will be used to record brain electrical activity. Measures will include EEG rhythms (α-waves: 8–12 Hz, reflecting a state of cerebral relaxation and correlating with cognitive function; β-waves: 12–30 Hz, associated with motor preparation/execution and serving as an indicator of neuroplasticity), Event-Related Potentials (ERPs: where P300 latency reflects cognitive processing speed, and P300 amplitude reflects attention engagement), Motor-Related Potentials (MRPs: which evaluate neural signals related to motor intention), and motor imagery ability (detected via BCI, reflecting the potential for motor function recovery). Together, these measures enable real-time monitoring of taVNS-induced activation of the cerebral motor cortex.
b. fNIRS-Related Metrics: This non-invasive method will measure regional cerebral blood flow (rCBF). Assessments will include cerebral metabolic activity (inferred from changes in oxyhemoglobin and deoxyhemoglobin concentrations in the prefrontal cortex and motor cortex, where reduced concentrations may indicate impaired brain function) and brain region activation patterns (evaluated through the hemodynamic responses of specific brain regions during cognitive tasks to assess prefrontal executive function and motor cortex coordination, with abnormal patterns suggesting neurological dysfunction). This approach allows for non-invasive monitoring of taVNS-mediated improvements in postoperative brain function.

#### 3.6.2 Secondary outcome measures

① Inflammatory Markers: Serum IL-6 and TNF-α levels will be measured via enzyme-linked immunosorbent assay. IL-6 is a key pro-inflammatory cytokine involved in systemic inflammation, and elevated levels correlate with vascular wall damage and organ dysfunction. TNF-α induces vascular endothelial cell apoptosis and promotes inflammatory cytokine cascades, with its levels positively correlating with the severity of postoperative inflammation in AD patients. Both markers will be used to evaluate the anti-inflammatory effect of taVNS.

② Neurotransmitters: Serum levels of 5-HT and BDNF will be measured via enzyme-linked immunosorbent assay. 5-HT is a critical neurotransmitter in both the central and peripheral nervous systems, involved in mood regulation and intestinal motility; reduced levels are associated with anxiety, depression, and gastrointestinal dysfunction. BDNF promotes neuronal survival, synaptogenesis, and neuroplasticity, and is closely linked to cognitive function and neural repair; low levels indicate impaired neural regeneration. These neurotransmitters will be used to evaluate taVNS-mediated regulation of the neuroendocrine system.

③ Intestinal Function: Bowel sound frequency will be recorded by auscultating periumbilical sounds every 4 h (normal range: 4–5 times per minute; reduced frequency indicates slowed intestinal peristalsis, while hyperactivity suggests intestinal irritation). The time to first postoperative spontaneous flatus and defecation will also be recorded (delays indicate impaired intestinal function recovery and serve as a direct marker of gastrointestinal dysfunction). These parameters will be used to comprehensively assess postoperative intestinal motility and function in AD patients.

④ Scale Scores: GIDS includes five items (e.g., abdominal distension, dyschezia) and has a total score ranging from 0 to 12, with higher scores indicating more severe gastrointestinal dysfunction. It will be used to assess the severity of gastrointestinal dysfunction. The HAMA consists of 14 items with a total score of 0–56 (a score ≥7 indicates the presence of anxiety, and ≥14 indicates significant anxiety). It will assess postoperative anxiety severity. The HAMD has 17 items and a total score of 0–52 (a score ≥7 suggests possible depression, ≥14 indicates moderate depression, and ≥24 indicates severe depression). It will assess postoperative depression. The SF-36 covers eight dimensions (e.g., physical function, mental health), with higher scores indicating better quality of life. It will evaluate quality of life. The PSQI includes seven components (e.g., sleep quality, sleep latency) and has a total score of 0–21 (a score >5 indicates poor sleep quality). It will assess postoperative sleep quality.

### 3.7 Safety evaluation

Throughout the study, participants' vital signs will be continuously monitored, and laboratory investigations—including complete blood count, urinalysis, coagulation profile, and hepatic/renal function—will be performed. An Adverse Event (AE) is defined as any unfavorable medical occurrence following taVNS administration, irrespective of its causal relationship to the intervention. AEs may manifest as new symptoms, signs, diseases, or laboratory abnormalities. All AEs will be actively monitored, documented in the CRF, and assessed for their nature, severity (Grades 1–4), and relationship to taVNS (definitely, probably, possibly, or unrelated).

A SAE is defined as any AE that results in death, is life-threatening, necessitates hospitalization or prolongs existing hospitalization, results in significant disability or incapacity, or is a congenital anomaly. Any SAE must be reported to the Ethics Committee of Guangdong Provincial Hospital of Traditional Chinese Medicine within 24 h, accompanied by a completed SAE report form.

### 3.8 Quality control

① Standardization of Personnel and Operations: All research staff will receive standardized training and must pass competency assessments to ensure consistent execution of taVNS procedures, scale evaluations, and biospecimen collection.

② Quality Control of Equipment and Testing: Standard Operating Procedures will be strictly adhered to. taVNS devices will be calibrated weekly, with stimulation intensity set to a perceptible but non-painful level. All laboratory assays will use approved reagents, and gut microbiota analysis will be conducted by an accredited third-party laboratory. Blood and stool specimens will be stored at −80 °C within one hour post-collection under unique study identifiers.

③ Data and Monitoring Management: Source data will be entered into the CRF and verified by two independent personnel. Independent monitors will audit ≥30% of cases bi-weekly to ensure data completeness and consistency across intervention records, laboratory results, and AE documentation.

④ Graded Handling of AEs: Grade 1 AEs will be managed on-site and reported within 24 h; Grade 2 AEs will lead to temporary intervention suspension with reporting within 72 h; Grade 3+ AEs will result in immediate intervention cessation and rescue measures, with reporting to the EC within 2 h. All study documents will be archived for 5 years to ensure traceability.

### 3.9 Data analysis and management

#### 3.9.1 Data collation

Data will be collected using CRFs, capturing baseline characteristics, intervention records, laboratory results, and scale scores. All biological specimens will be de-identified via unique codes to protect participant privacy. Statistical analyses will be performed using SPSS 22.0, with the following approach:

① Statistical Tests by Data Type: Categorical variables will be summarized as frequencies (percentages) and compared using the chi-square test. Continuous variables will be presented as mean ± standard deviation; after testing for normality and homogeneity of variance, parametric data will be analyzed using *t*-tests (independent or paired, as appropriate), while non-parametric data will be analyzed using the Mann–Whitney *U* test (rank-sum test). Ordinal data (e.g., scale scores) will also be analyzed using non-parametric rank-sum tests.

② Analysis of Primary and Secondary Outcomes: Outcomes include scale scores (GIDS, HAMA, HAMD, SF-36, and PSQI), brain function parameters (EEG, fNIRS-derived rCBF), gut microbiota metrics (diversity indices, taxon abundance), and biochemical markers (IL-6, TNF-α, 5-HT, and BDNF). A stratified multiple comparison adjustment will be applied: the primary outcome will use Bonferroni correction (corrected α = 0.01, accounting for five indicators), while secondary outcomes and biomarkers will use the False Discovery Rate (FDR) method (FDR = 0.05). Subgroup analyses (e.g., by gender, Stanford type) will also employ FDR correction to control Type I error inflation.

③ Correlation Analysis: Multiple linear regression will be used to explore associations between clinical outcomes, gut microbiota, inflammatory markers, and brain function.

④ Significance Level: A two-sided *P* value < 0.05 will be considered statistically significant for all tests, unless otherwise specified after multiplicity adjustment.

#### 3.9.2 Main statistical models

Mixed-effects models will serve as the core analytical framework to handle repeated measures collected at 1 day, 7 days, 4 weeks, and 24 weeks postoperatively. ① A linear mixed-effects model will be used for continuous outcomes, including gut microbiota indices (Shannon index, Bifidobacterium abundance), brain function parameters (fNIRS rCBF, EEG α-wave power), and biochemical markers (IL-6, TNF-α, 5-HT, and BDNF). ② A logistic mixed-effects model will be applied to binary outcomes, such as adverse events (SAE, skin allergy) and intestinal function recovery (first flatus within 48 h, first defecation within 72 h). ③ An ordinal logistic mixed-effects model will be used for ordinal outcomes, including scale scores (GIDS, HAMA, and HAMD).

#### 3.9.3 Handling missing data

The pattern of missing data will be first assessed using Little's test for Missing Completely at Random (MCAR). The mechanism will be classified as: ① MCAR: *P* > 0.05, indicating missingness is independent of both observed and unobserved data. ② Missing at Random (MAR): *P* < 0.05, but missingness is explainable by observed variables. ③ Missing Not at Random (MNAR): Missingness depends on unobserved data, as inferred from investigator notes and adverse event logs.

For data assumed to be MCAR or MAR, multiple imputation will be performed. The imputation model will include all outcome variables, baseline characteristics, and potential confounders. Predictive mean matching, logistic regression, and proportional-odds models will be used to impute continuous, binary, and ordinal data, respectively. Results from five imputed datasets will be pooled using Rubin's rules. For data suspected to be MNAR, a complete case analysis (CCA) will be the primary method, supplemented by sensitivity analyses to assess the robustness of the findings. Should the CCA and sensitivity analyses yield discrepant results, the potential impact of MNAR will be discussed as a study limitation. An interim review will be triggered if the missing rate for any outcome exceeds 30%, or if the rate for a key primary outcome surpasses 20%, to reassess data collection feasibility.

## 4 Discussion

The aim of this study is to explore the clinical efficacy and potential mechanisms of taVNS in treating postoperative complications of AD by regulating the GBA, using a randomized controlled trial design. AD is a life-threatening cardiovascular emergency, and postoperative complications such as systemic inflammatory response syndrome, gastrointestinal dysfunction, anxiety, and depression significantly affect patient prognosis. Existing treatment options have a limited capacity for the synergistic management of such multi-system disorders (20). The GBA is a bidirectional communication system involving key components including gut microbiota, the intestinal barrier, the nervous system, and the endocrine system. It plays a comprehensive role in neuroregulation, immune regulation, and metabolic regulation at multiple levels—such as neuroinflammation, endocrine disorders, and immune responses—during the postoperative period of AD (21, 22). Based on the central role of the GBA within the neuro-endocrine-immune network, this study innovatively applies taVNS, a non-invasive neuromodulation technique, to the postoperative management of AD. This approach aims to provide a new therapeutic strategy for the multi-target intervention of complex complications.

Existing studies have confirmed that the pathological process of AD is closely related to GBA dysregulation. The team of Zheng Shuai et al. (23) studied 40 patients with gastrointestinal dysfunction following Type A aortic dissection and found that postoperative levels of inflammatory markers—including white blood cell count, neutrophil count, IL-2, IL-6, IL-8, and IL-10—were significantly elevated. Additionally, the abundance of gut microbiota such as Oscillibacter, Anaerotruncus, Alistipes, and Clostridium was increased; these changes may be key factors contributing to gastrointestinal dysfunction after AD. Meanwhile, a series of studies by Wang Yapeng's group on perioperative brain function monitoring in Type A aortic dissection patients (24–26) have shown that quantitative electroencephalography is an effective tool for evaluating and predicting neurological outcomes after AD surgery. Using quantitative electroencephalography parameters such as dynamic amplitude-integrated electroencephalography grading and postoperative relative band power of the α rhythm, they found these parameters to be closely associated with adverse postoperative outcomes and transient neurological dysfunction, and they could serve as independent risk factors. These studies highlight the importance of perioperative gut microbiota analysis and brain function monitoring in managing AD postoperative complications, which together constitute the regulatory targets of GBA dysfunction following AD surgery.

Although recent studies on taVNS have confirmed its anti-inflammatory, intestinal regulatory, and neurotransmitter-balancing effects in common postoperative conditions such as insomnia (27), anxiety and depression (28), constipation (29), cognitive impairment (30), and postoperative subarachnoid hemorrhage (31), its application in AD postoperative complications has not been reported. This study employs primary outcome measures—including gut microbiota diversity and abundance, BCI-related indicators, and fNIRS-related indicators—as well as secondary measures such as inflammatory factors, neurotransmitters, intestinal function, and relevant scale scores to determine the efficacy and mechanism of taVNS in regulating the GBA for treating AD postoperative complications. In a mouse model of constipation-predominant irritable bowel syndrome, taVNS has been shown to restore *Lactobacillus* abundance, increase probiotics like *Bifidobacterium*, and improve defecation function, gastrointestinal transit, and visceral hypersensitivity; the regulation of gut microbiota is an important mechanism underlying these effects (13). Combined with EEG analysis of BCI-related indicators, studies have found that taVNS can improve learning performance, reduce the amplitude of movement-related cortical potentials and the α–γ modulation index, and enhance frontal lobe functional connectivity. Furthermore, EEG reveals that taVNS improves motor learning by enhancing cognitive memory (32). fNIRS can detect that taVNS improves accuracy in the No-Go task (which requires inhibition of responses to specific stimuli), reduces information accumulation in the Go task (which requires responses to target stimuli), enhances engagement of the bilateral inferior frontal gyrus and prefrontal network connectivity, and improves emotional inhibition. Thus, fNIRS helps elucidate the neural mechanisms of taVNS (33). Previous studies have shown (34) that taVNS can ameliorate depression-like behaviors in a lipopolysaccharide (LPS)-induced rat model of inflammatory depression, regulate peripheral pro-inflammatory factors (e.g., IL-1β, TNF-α) and anti-inflammatory factors (e.g., IL-4, IL-10), and inhibit activation of the NF-κB pathway in the prefrontal cortex, thereby playing a key role in modulating the peripheral-central inflammatory network. taVNS is thought to exert its effects through the vagus nerve's “cholinergic anti-inflammatory pathway.” Specifically, its anti-inflammatory effect at 15 Hz is superior to that at 25 Hz: it can reduce levels of inflammatory factors, immune cell infiltration, and tissue damage, showing feasibility for treating acute inflammation (35). Additionally, taVNS's regulation of central neurotransmitters (5-HT and BDNF) may alleviate postoperative anxiety and depression and improve cognitive function, thereby interrupting the vicious cycle of psychological stress and abnormal vascular tone (36, 37). Scores from multidimensional scales, including the GIDS, HAMA, HAMD, SF-36, and PSQI, can also reflect the improvement of various clinical symptoms and the interventional efficacy in postoperative AD patients.

The innovations of this study are reflected in three aspects: First, the combination of taVNS and the GBA theory is applied to the postoperative management of AD for the first time, overcoming the limitations of traditional single-system interventions by regulating complications through the synergistic actions of neural, immune, and endocrine pathways. Second, a multidimensional outcome indicator system is utilized, which includes not only core GBA biomarkers such as gut microbiota diversity and inflammatory factors but also, through collaboration with the rehabilitation department, real-time monitoring of brain function changes via BCI and fNIRS, enabling cross-system correlation analysis of intestinal function, inflammation, and brain function in postoperative patients. Third, a randomized controlled design is strictly followed: a sham stimulation control (with no current output, targeting non-vagus nerve innervated areas) is implemented, and stratified randomization based on the Stanford classification is employed to reduce bias and enhance the reliability of the results.

From a methodological perspective, the intervention protocol in this study is rigorously designed. For taVNS, the stimulation sites are selected as the cymba conchae or tragus (areas rich in the auricular branches of the vagus nerve), and the parameters are set to a frequency of 15 Hz and a pulse width of 200 μs—based on international standards and previous anti-inflammatory research evidence—to ensure the stimulation intensity is within patients' tolerance range while effectively activating the vagus nerve pathway. The sample size estimation is based on effect sizes of gut microbiota and inflammatory factor changes reported in similar studies; after accounting for a 10% dropout rate, 25 cases per group were determined, meeting statistical power requirements. The procedures for collecting and processing biological specimens (e.g., fecal samples stored at −80 °C, metagenomic sequencing for gut microbiota structure analysis) are standardized, and analyses are conducted by third-party laboratories to ensure data objectivity.

However, this study has several limitations: First, the single-center design may limit the generalizability of the results, as regional patient characteristics (e.g., distribution of underlying diseases, differences in surgical approaches) could influence treatment efficacy. Second, due to various constraints, the sample size is relatively small, resulting in insufficient statistical power for subgroup analyses—including analyses of rare adverse events and efficacy differences between Stanford Type A and Type B patients. Third, implementing blinding poses challenges: although the sham stimulation group uses devices identical in appearance, the subtle sensation from real stimulation might lead to unblinding of participants, potentially affecting the interpretation of results. Fourth, the 24-week follow-up period may be insufficient to fully capture the long-term effects of taVNS on prognostic outcomes in AD patients, such as aortic remodeling and reoperation rates.

Despite these limitations, the potential value of this study is noteworthy. If taVNS is proven effective, it could provide a safe and cost-effective non-pharmacological treatment for managing postoperative complications in AD patients, being particularly suitable for multi-system regulation during the long-term rehabilitation phase. From a mechanistic perspective, this study may reveal the regulatory role of the GBA in postoperative complications of cardiovascular diseases and provide new evidence for cross-system interactions among the neural, immune, and intestinal systems.

Building on this study, future research should include prospective, multi-center, large-sample trials and extend follow-up durations to better evaluate long-term prognosis. Additionally, animal models could be used to further explore the specific regulatory mechanisms of taVNS on vagus nerve pathways and gut microbiota metabolites, laying the groundwork for precise targeted interventions. In summary, by integrating taVNS technology and the GBA theory, this study offers innovative insights for the multidimensional management of postoperative complications in AD. Its results will provide evidence-based support for clinical practice and promote the application and development of neuromodulation techniques in multi-system diseases.
